# Chloroform in Labor

**Published:** 1856-04

**Authors:** 


### CHLOROFORM IN LABOR.

Below will be found some interesting extracts from the records of the Cincinnati Medical Society, published in the Western Lancet, and containing some pertinent and appropriate observations in reference to the Ethics, which should belong at all times to a Medical Society, with a report and discussion upon the use of Chloroform in labor, in relation to the propriety of which there has been so much discussion and such a wide difference of opinion among the profession.

We have been long satisfied that in *ordinary labor*, its use was wholly unjustifiable, upon the ground that it is an interference with a natural process, by the substitution of a dangerous agent, for the pain, which the Creator has attached to the performance of the function, a freedom from which, in our view, is a questionable benefit, even if it could be obtained at half the risk attending the plan proposed.

We have no hesitation—with the present and daily developing light, in reference to the dangerous character of this agent—in asking the question, Who can tell in what case the *inhalation* of Chloroform, to Anæthesia, is entirely free from danger? and though we are not prepared to question its value in surgery or difficult obstetrics, and upon the whole look upon it as an important addition to the Materia Medica, we would not experience the horror attending the death of a female in *natural labor*, where it had been used by our instrumentality, for a right arm.

And we entirely accord with the views intimated in the report of Dr. Walker, as to the construction which should be placed upon the statistics presented by Dr. Burwell, of its use in the Buffalo Hospital, and it is rather surprising to us, that the reviewer of such statistics should have come to the conclusion, that these most remarkable results could not, with the

*slightest degree of probability*, be attributed to the use of Chloroform.

*Extract from the Annual Report to the Cincinnati Medical Society, for* 1855, *furnished by* Dr. Walker.

"Anæthesia has been used to some extent the past year in obstetrics by several of our physicians, and is, I believe, gaining many advocates. That in some *rare* cases where the symptoms are such as to require the aid of art to complete delivery, its use is justifiable, there can be but little doubt; but its indiscriminate use is, to say the least, questionable. Labor is a normal function, and is usually attended with no danger to mother or child. Anæsthetic agents have been instrumental in the immediate death of more than one hundred persons as shown from statistics, some of whom even in the child-bed room. Should we not then be exceedingly cautious how we interfere with one of *nature's laws*, through an uncertain, and perhaps a deadly agent?

"Here it may be well to present a few facts from statistics, to the society, taken from the review of Dr. Burwell's Essay of the Buffalo Hospital, by Prof. Barker, of New York. Both the essayist and the reviewer are favorable to its use, therefore we should expect the review to be a fair one.

"That I may do Dr. Burwell no injustice, I remark that I have not been able to obtain a copy of his essay, but merely the review. I have taken for granted that these were promiscuous cases taken as they presented themselves at the Hospital.

| | |
|---|---|
| "'Whole number of cases reported | 180 |
| Number of cases requiring forceps | 17 |
| " " " Craniotomy | 1 |
| " " " Turning | 1 |
| Still born children | 14 |
| Flooding | 7 |
| *Bad health succeeding* | 25.' |

"Prof. Barker says that it is satisfactorily established by the Essayist, that neither the death of a child—the inflammation, flooding, or bad health succeeding, can with the *slightest degree of probability* be ascribed to the use of chloroform.

"Is it *possible* to explain why forceps should be used once in ten and a half cases? That one of every 13 children should be born dead? Or, that one in every seven women should have bad health following child-birth?

"It may be explained to *his* satisfaction; but if such results should follow even *your* practice, Mr. President, as skilled as you are in that department, you would soon find that the community in which you live, could not be made to understand the propriety of your services. For my own part, if I was satisfied that one in seven of my patients must suffer bad health after confinement, I would forever abandon the profession.

"That the use of these agents have a direct tendency to produce the very results above named, there can be no doubt, from the testimony of its friends. It stimulates to depress—it retards labor, and Professor Barker says '*he* would not give it in face or breech presentations when a delay might be dangerous.'

"One case during the past year has been related, intending to show the superiority of opiates over the use of ergot, or the forceps, when from exhaustion, the pains of labor cease, though the case mentioned would not be conclusive; still it may be well to consider carefully whether rest would not be more in consonance with nature's laws, than the attempt to excite with ergot, or to remove the child by mechanical force.

"Our Society has now been in existence four years. The necessity for its existence, and the correctness of its basis, is no longer a matter of question. That it has done much to harmonize the profession of our city, there is no doubt. We have no written code of ethics to govern us, it is true; but we trust a code *is* written on the heart of each of our members—the code of gentlemen.

"Our meetings have been more interesting and profitable, as during the whole time nothing foreign to scientific and medical subjects have been entertained; thus excluding all topics which would seem to engender strife and personalities, too common attendants on medical societies. Allow me to suggest the propriety of shorter essays, and for us to devote more time to the relation and discussion of cases. Many of our essays are very interesting and important, but do not bring out so general a discussion as would be profitable in a society of this kind. They are better suited to the pages of a Journal.

Should we devote more time to the discussion of cases, we should have before us more practical matter, and with a good reporter at our table, we should spread upon our minds and before the world, much of interest to the profession, which otherwise would be forever lost.

"In order for a full and and free relation of cases here, however, it is all important that there should be a perfect feeling of confidence in each other. Our relations in a medical society should of right be, as sacred as that which exists in the relation we bear to a patient—a relation of *perfect confidence;* without it no person in his right mind would relate a case of any delicacy, though no names were given; and no person unless a madman or fool, would take advantage of such relation for his own advancement, or the injury of his medical brother."

---

### DISCUSSION.

"Dr. Bonner spoke highly of the report, and moved its publication. As regards the use of chloroform, he did not think that any man of common sense would use it in cases of natural labor; he would even go so far as to say its use in such cases was deserving of legal prosecution. Doubtless it was sometimes necessary, so were the forceps and crotchet, and with those it should be classified.

"Dr. Wright did not know whether the statistics presented by Dr. Walker were intended to show a favorable or unfavorable result from the use of chloroform. He would take it for granted, however, that it was the latter. When fairly considered, it will appear to every body that the success as represented by the report, has been remarkable. He shows a list of 180 cases in which it had been administered, and amongst these, 40 cases were attended with dangerous complications, and in only 25 cases did bad health follow, nor does he say to what this bad health amounted. He would ask if any statistics could be furnished, showing results equally satisfactory without its use. It will be admitted by all that the application of the forceps at the superior strait is attended with a great deal of difficulty, sometimes from the nervous restlessness of the patient, and the spasmodic action of the muscles; by the administration of chloroform, she is rendered as manageable as a manikin in the hands of her attendant, rendering the application of the instrument more easy and safe. Indeed few cases present, where the forceps are required at the superior strait, in which chloroform is not absolutely demanded. Professor Simpson, of Edinburgh, has used it in many thousand cases, and only in one case has death occurred—one hundred ounces have been administered by Dr. Simpson, in the space of three weeks, to a child of five weeks old suffering from convulsions, with perfect safety to the child, and cure of the disease. If then no danger has resulted from its use, why deprive the sufferer of its benefit? Dr. W. has used it indiscriminately in the two-thirds of all his cases, and has yet to learn of any unpleasant results following its administration, even in cases where difficulties had previously occurred without its use. As to fear of any danger of its proving injurious, he would have less hesitancy than from the use of opium or any active narcotic, it being more under the control of the physician; indeed, there is no remedy in the pharmacopœia more under control than chloroform. When, therefore, the patient wishes for it, he offers no objection to it.

"Dr. Bonner did not object to the use of chloroform when interference with instruments was necessary, or even where the woman was suffering from irregular or harassing pains; but to its being given indiscriminately, and in cases of natural labor, he looked upon it as interfering with the laws of God and nature. That danger does attend its use, history will afford sufficient proof; even our own city can furnish instances of its fatal effects.

"Dr. Walker wished Dr. Wright would inform him what the symptoms were which would render its use unadvisable or stop its administration.

"Dr. Wright has had no occasion to stop its administration, nor have any bad symptoms occurred under his notice.

"Dr. Walker asked if it did not retard labor.

"Dr. Wright thinks it rather expedites it.

"Dr. Comegys asked if Dr. Wright apprehended any danger to the child in utero, from the prolonged use of chloroform, as he thinks he heard him remark so at one time.

"Dr. Wright said not. As a precaution he had (in the case alluded to) the child removed as soon as possible from the room which was largely impregnated with the vapor of chloroform.

"Dr. Comegys asked if hemorrhage was more liable to occur. He had noticed hemorrhage which he supposed was more profuse than would have been, had not chloroform been used.

"Dr. Wright did not think there was any more liability, nor has he seen any indications of an increase of hemorrhage.

"Dr. Wm. Wood has used it in some cases, such as Dr. Bonner alluded to; it seemed to him to retard the delivery. He agrees with Dr. Bonner in considering the action of labor a physiological process capable of fulfilling its intended end; with regard to hemorrhage he should differ with Dr. Comegys, as it appeared comparatively less in those cases under his knowledge. It is a question whether the patient is actually relieved from pain by the use of chloroform, as they seem to suffer just as much as when it has not been used; the same may be said of surgical operations, though the patient is afterwards unconscious of having suffered. The following coincidence may not be uninteresting. A lady who had been married eight years without having children, became pregnant the first time, after an attack of small pox; another, married three years, also became pregnant for the first time, after a similar attack; a third has recovered a short time since from small pox, who has been married eight years without children, with a probability of a similar occurrence.

"Dr. J. J. Quinn's opinion coincided with that of Dr. Bonner; during the past year he has administered it three times; in two cases where turning had to be resorted to, in both the child was dead, though the mother had not been under its influence more than an hour; the third, which was a case of very difficult and tedious labor, both mother and child died. Last week had a case of shoulder presentation, turning was resorted to without chloroform, and the child was born alive."

